# Illumina MiSeq sequencing reveals the effects of grape seed procyanidin on rumen archaeal communities *in vitro*

**DOI:** 10.5713/ajas.19.0226

**Published:** 2019-07-01

**Authors:** Hua Zhang, Jinjin Tong, Zun Wang, Benhai Xiong, Linshu Jiang

**Affiliations:** 1 Beijing Key Laboratory of Dairy Cow Nutrition, Beijing University of Agriculture, Beijing 102206, China; 2 State Key Laboratory of Animal Nutrition, Institute of Animal Science, Chinese Academy of Agricultural Sciences, Beijing 100193, China

**Keywords:** Grape Seed Procyanidin Extract, Archaeal Community, Methanogenesis, MiSeq High-throughput Sequencing

## Abstract

**Objective:**

The present study explored the effects of grape seed procyanidin extract (GSPE) on rumen fermentation, methane production and archaeal communities *in vitro*.

**Methods:**

A completely randomized experiment was conducted with *in vitro* incubation in a control group (CON, no GSPE addition; n = 9) and the treatment group (GSPE, 1 mg/bottle GSPE, 2 g/kg dry matter; n = 9). The methane and volatile fatty acid concentrations were determined using gas chromatography. To explore methane inhibition after fermentation and the response of the ruminal microbiota to GSPE, archaeal 16S rRNA genes were sequenced by MiSeq high-throughput sequencing.

**Results:**

The results showed that supplementation with GSPE could significantly inhibit gas production and methane production. In addition, GSPE treatment significantly increased the proportion of propionate, while the acetate/propionate ratio was significantly decreased. At the genus level, the relative abundance of *Methanomassiliicoccus* was significantly increased, while the relative abundance of *Methanobrevibacter* decreased significantly in the GSPE group.

**Conclusion:**

In conclusion, GSPE is a plant extract that can reduce methane production by affecting the structures of archaeal communities, which was achieved by a substitution of *Methanobrevibacter* with *Methanomassiliicoccus*.

## INTRODUCTION

Procyanidins, a group of flavonoids, are oligomeric forms of catechins that are abundant in red wine, grapes, cocoa, tea and apples and are thought to have beneficial effects on health. It has been reported that grape seed procyanidin extract (GSPE) exhibits many biological activities, such as free radical scavenging, antitumor activity, antioxidative stress activity and other biological activities. Furthermore, procyanidins function as powerful antioxidants and can have anti-inflammatory effects [1]. Li et al [2] reported that procyanidins (of larch bark) showed antibacterial effects and mechanisms on *Staphylococcus aureus* by destroying the integrity and permeability of cell wall/membrane, affecting protein synthesis, and binding to DNA grooves to form complexes. The improvement of livestock production performance by the addition of plant extracts has broad application potential, especially for the regulation of productivity and mitigation of methane production in dairy cows. Gessner et al [3] reported that dairy cows fed grape seed extract during the transition period significantly increased milk yield. Similarly, Correddu et al [4] found that grape seed could help increase the concentration of milk fatty acids with potential health benefits in dairy ewes.

The rumen is an anaerobic microbial ecosystem containing a dense mixture of protozoa, bacteria and anaerobic fungi that convert carbohydrates into short-chain volatile fatty acids (VFAs), which are absorbed by the animal and used in energy metabolism and protein synthesis. Methane is a natural product of anaerobic microbial fermentation and is accompanied by a significant loss of feed efficiency [5]. The fermentation process is performed by a group of archaea collectively known as methanogens, which belong to the phylum Euryarchaeota. Hydrogen is formed during fermentation and is used by methanogenic archaea to reduce CO_2_ to methane. Ungerfeld [6] found that maximizing the metabolic H_2_ away from CH_4_ and toward propionate formation would increase the efficiency of ruminant performance and reduce the environmental impact. Moreover, methanogenesis from ruminants depends in part upon the abundance of methanogenic archaea and/or H_2_-producing microorganisms [7]. Therefore, shifting the archaea community may be an effective measure to promote feed energy utilization.

The objective of the present study was to investigate the potential effects of grape seed procyanidins on propionate production, methanogenesis and the archaeal community *in vitro* via Illumina MiSeq sequencing.

## MATERIALS AND METHODS

### Experimental design and *in vitro* batch incubation

This study was performed at the Key Laboratory for Dairy Cow Nutrition, College of Animal Science and Technology, Beijing University of Agriculture, Beijing, China. The experimental methods were approved by the Animal Care and Use Committee of Beijing University of Agriculture and were in compliance with the Regulations for the Administration of Affairs Concerning Experimental Animals (The State Science and Technology Commission of P. R. China, 1988). The GSPE (90% procyanidins, which are members of the proanthocyanidin class of flavonoids) was purchased from Tianjin Jianfeng Natural Product R&D Co., Ltd. (Tianjin, China).

This experiment was conducted according to a completely randomized design to identify the effects of GSPE on gas production, fermentation patterns and the archaeal community. There were two treatments: the control group (group A, CON, no GSPE adding; n = 9) and the treatment group (group B, GSPE, 1 mg/bottle GSPE, 2 g/kg dry matter [DM]; n = 9). Fresh ruminal fluid was collected from four cannulated lactating Holstein dairy cows (670±24 kg body weight; 114.6±7.5 days in milk) 2 h after the morning feeding, mixed in equal amounts, and poured into a sterilized bottle such that no headspace remained in the bottle. The bottle was brought to the laboratory within 30 min. The ruminal fluid was extruded into the flask through four layers of gauze, and the flask was filled with CO_2_ and placed in a 39°C water bath until use. The fresh ruminal fluid was diluted with buffer solution (1:2 v/v), which was prepared as described by [8]. The incubation progress *in vitro* was operated as we previously described [9]. Briefly, 75 mL of inoculum-buffer mixture was added to each 120-mL serum bottle, which contained 500 mg of mixed diet DM as fermentation substrates. The composition of the mixed diet is shown in Table 1. The GSPE (1±0.1 mg) was measured in advance using analytical balance (ME204, Mettler Toledo, Uster, Switzerland) and dosed into the treatment group serum bottles. All bottles were sealed with crimp-sealed rubber stoppers to prevent gas leakage and connected with vacuumed air bag (Hedetech, Dalian, China), then affixed to a rotary shaker platform (90 rpm) in a temperature-controlled (39°C±0.5°C) incubator (THZ-C, Taichang, Shijiazhuang, China) for 24 h. This experiment was repeated in three batches, and three units per treatment were arranged in each batch.

### Sampling and chemical analysis

The total gas production was measured using a 100-mL calibrated glass syringes (Häberle Labortechnik, Lonsee-Ettlenschieß, Ettlenschiess, Germany). Gas samples (5 mL) were used to detect the concentrations of methane and hydrogen by gas chromatography (7890B, Agilent Technologies, Foster City, CA, USA). The productions of methane and hydrogen were calculated from the methane and hydrogen concentration and total gas production according to ideal gas law (pV = nRT). The pH value of each fermentation fluid was measured with a pH meter (SevenGo TM pH meter SG2, Mettler Toledo, Switzerland). VFA analysis was conducted on 1 mL of each fermentation fluid, which was preserved by adding 0.2 mL of 25% H_3_PO_4_ by gas chromatography (7890B, Agilent Technologies, USA), as reported by Mao et al [10]. All the samples were stored at −80°C until the analyses. The pre-weighted filter bags (ANKOM Technology, Macedon, NY, USA) were used to filter the fermentation fluid of each serum bottle. Then the filter bags were washed with cold running water until the effluent ran clear. At last, the filter bags were dried at 55°C for 48 h for analysis of the DM digestibility.

### DNA extraction

Microbial DNA extraction was carried out on the rumen samples with cetyltrimethylammonium bromide and bread-beating methods as previously described by Jin et al [11]. DNA quality was determined by 1% agarose gel electrophoresis containing Goldview TM (Saibaisheng, Shanghai, China). The yield and purity of the extracted DNA were assessed with a NanoDrop 1000 instrument (NanoDrop, Wilmington, DE, USA).

### Real-time polymerase chain reaction analysis

The DNA samples were used as templates to quantify the total bacterial abundance, and the abundances of anaerobic fungi, ciliate protozoa, methanogens, *Fibrobacter succinogenes*, *Ruminococcus albus*, and *Ruminococcus flavefaciens* were assessed. All primers and assay conditions used in this study have been previously published [12]. Each reaction involved 3 duplicates amplified using a Step-One Plus real-time polymerase chain reaction (RT-PCR) system (Applied Biosystems, Foster City, CA, USA) with SYBR *Premix Ex Taq* (Takara, Dalian, China) and were performed according to previously published methods [13]. Changes in the targeted populations were calculated using a relative quantification calculation, and the total bacterial Ct (cycle threshold) value was used as the reference value: target bacterial population (%) = 100× 2^−(Ct target – Ct total bacterial)^. Changes in microbial communities due to the addition of GSPE are expressed as a percentage change relative to the control population.

### 16S rDNA analysis

The V3-V4 regions of the archaeal 16S rRNA gene were amplified by PCR (95°C for 3 min; followed by 27 cycles at 95°C for 30 s, 55°C for 30 s, 72°C for 45 s; and a final extension at 72°C for 10 min) using the primers 349F (5′-barcode-GYGC ASCAGKCGMGAAW-3′) and 806R (5′-GGACTACVSGG GTATCTAAT-3′) [14], where the barcode is an eight-base sequence that is unique to each sample. The PCRs were performed in triplicate in a 20-μL mixture containing 4 μL of 5× FastPfu buffer, 2 μL of 2.5 mM dNTPs, 0.8 μL of each primer (5 μM), 0.4 μL of FastPfu polymerase, 0.2 μL of bovine serum albumin and 10 ng of the template DNA. Amplicons were excised from 2% agarose gels, purified using the AxyPrep DNA Gel Extraction Kit (Axygen Biosciences, Union City, CA, USA) according to the manufacturer’s instructions, and quantified using a QuantiFluor-ST instrument (Promega, Madison, WI, USA). Finally, purified amplicons were pooled in equimolar ratios and subjected to paired-end sequencing (2×300) on an Illumina MiSeq platform according to standard protocols. The raw reads were deposited into the NCBI Sequence Read Archive database (accession number: SRP139902).

### Sequencing data processing and analysis

Sequencing data processing and analysis followed our previously described procedures [15]. Briefly, the raw FASTQ files were quality filtered using Trimmomatic software and merged using FLASH with the following criteria: i) the reads were truncated at any site that received an average quality score of <20 over a 50-bp sliding window; ii) sequences with overlapping segments longer than 10 bp were merged according to their overlapping sections with a mismatch of no more than 2 bp; iii) the sequences of each sample were separated based on the barcodes (exactly matched) and primers (allowing 2-nucleotide mismatches), and reads containing ambiguous bases were removed. Subsequently, the average length of all the clean reads was 372 bp and the average sequencing depth was 52,281 clean reads for archaeal community analysis. Operational taxonomic units were clustered at 97% sequence identity using UPARSE [16]. The ribosomal database project was used for the taxonomic classification of the sequence. Simpson, Shannon, Chao, and ACE indices were calculated for each sample. LEfSe analysis was carried out using R-3.2 with the vegan package on the online Majorbio I-Sanger Cloud Platform (http://www.i-sanger.com) according to our previously reported methods [15].

### Statistical analysis

Data for gas production, NH_3_-N, DM digestibility, microbial crude protein, CH_4_, H_2_, ruminal pH, VFA concentrations and the alpha diversity index were analyzed using PROC MIXED of SAS 9.4 (SAS Institute, Inc, Cary, NC, USA), as shown in the following model: Y_ijk_ = μ+T_i_+B_j_+TB_ij_+e_ijk_, where Y_ijk_ is the dependent variable, μ is the overall mean, T_i_ is the effect of treatment (GSPE or CON, considered fixed), B_j_ is the effect of batches (j = 1,2,3, consider fixed), TB_ij_ is the ineraction between T_i_ and B_j_ (considered fixed) and e_ijk_ is the residual. The archaeal abundance was log 10 (n+1) transformed to ensure normal distribution. Statistical significance is defined when p values are less than 0.05, and a trend was indicated by p<0.10.

## RESULTS

### Effects of grape seed procyanidin on gas production and volatile fatty acid

The fermentation results with grape seed procyanidin treatment are summarized in Table 2. The DM digestibility, gas production and methane production were significantly decreased by the addition of GSPE. The proportion of propionate significantly increased by the GSPE supplement, while the acetate to propionate ratio was decreased. Compared with the control group, the proportion of acetate exhibited a decreasing tendency. Overall, the results indicate that grape seed procyanidin had a significant impact on the VFA profile, especially on propionate formation.

### Effects of grape seed procyanidin on microbial populations

The RT-PCR results showed that the relative populations of methanogens, ciliate protozoa, and *Fibrobacter succinogenes* were significantly decreased by 52%, 42%, and 51%, respectively (Table 3). Compared with CON, the population of anaerobic fungi was increased by 50% in the grape seed procyanidin group, while *Ruminococcus albus* and *Ruminococcus flavefaciens* were not affected.

### Effects of grape seed procyanidin on archaeal community

**Figure 1:**
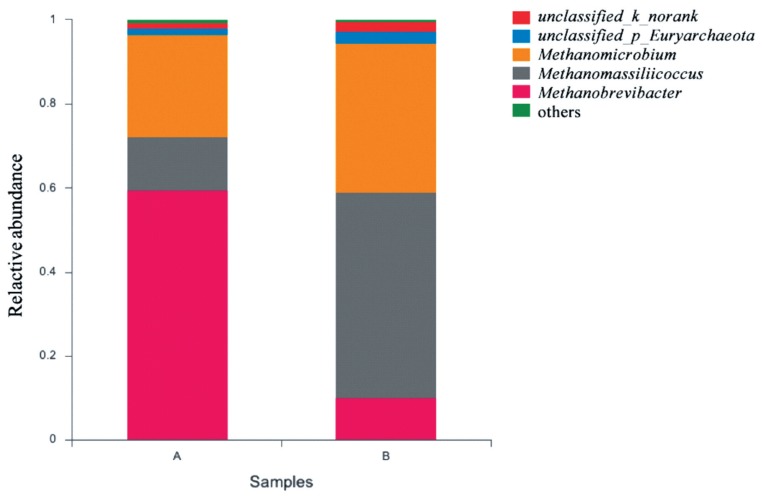
Composition of the predominant archaea genera among treatments *in vitro*. The top 5 abundant archaea genera are shown and the others are not shown. (A) Control group, no grape seed procyanidin extract addition. (B) Treatment group, 1 mg/bottle grape seed procyanidin extract addition (n = 9).

**Figure 2:**
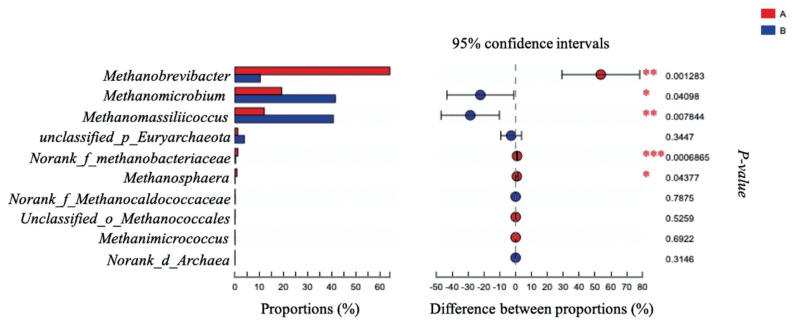
Difference in the relative abundance of archaeal genera (abundance of the genera was expressed as %). Extended error bar plot was performed by bioinformatics software (STAMP). Welch’s two-sided test was used and Welch’s inverted was 0.95. (A) Control group, no grape seed procyanidin extract addition. (B) Treatment group, 1 mg/bottle grape seed procyanidin extract addition (n = 9).

The effects of grape seed procyanidin on the alpha diversity of the ruminal archaeal community are summarized in Table 4. No significant differences were observed among the alpha diversity indices upon grape seed procyanidin treatment. The archaeal community was represented by the predominant phylum Euryarchaeota in both the grape seed procyanidin and control groups, comprising an average of 98% of the archaeal community. At the genus level, organisms with a relative abundance of ≥0.1% of the total sequences in at least one of the samples were further analyzed. The top 3 predominant genera were *Methanobrevibacter*, *Methanomicrobium*, and M*ethanomassiliicoccus* in both groups (Figure 1). The relative abundance of *Methanobrevibacter* was lower in the GSPE-treated group than that in the control group (Figure 2). Additionally, the abundances of *Methanomicrobium* and M*ethanomassiliicoccus* were significantly increased by the GSPE treatment.

### Biomarker genera within the microbial community

**Figure 3:**
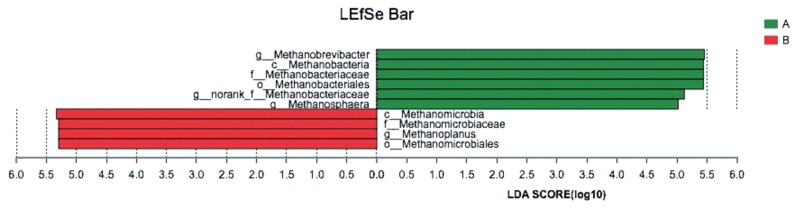
LEfSe analysis of the biomarker genera in the microbial communities in control group and treatment group. The LEfSe analysis combined the rank sum tests and taxonomic information to determine biomarker genera with the largest impact on community structure. (A) Control group, no grape seed procyanidin extract addition. (B) Treatment group, 1 mg/bottle grape seed procyanidin extract addition (n = 9).

The LEfSe analysis combined the rank sum tests and taxonomic information to determine biomarker genera with the largest impact on community structure. In our study, 4 methanogenics were selected as biomarkers for the control group, and 6 methanogenics were selected as biomarkers for the GSPE-treated group. A list of the biomarker genera is shown in Figure 3.

## DISCUSSION

Our results indicate that grape seed procyanidin has a significant effect on the VFA profile and methane production. Microorganisms are solely responsible for feed digestion and for the production of methane and VFA in the rumen. Previous studies have demonstrated that grape seed procyanidin has a positive effect on milk production [4,17], particularly via its effects on ruminal metabolism. Therefore, a comprehensive characterization of the microbial community richness and/or activity is essential to understand the effect of grape seed procyanidin on rumen fermentation and methane production. This study revealed, for the first time, the effect of grape seed procyanidin on archaeal groups using *in vitro* fermentation coupled with high-throughput sequencing. Moreover, this study also elucidated the potency of grape seed procyanidin as an additive for maintaining low methanogen levels and preventing negative effects while modulating rumen fermentation.

### Effect of grape seed procyanidin on methane production and related changes in archaeal communities

The present study indicated that there was a reduction in methane production after supplementation of grape seed procyanidin, followed by a significant decrease in the relative abundance of *Methanobrevibacter* and a significant increase in that of *Methanomassiliicoccus*. There were three major pathways for methanogenesis in rumen: hydrogenotrophic, methylotrophic and acetoclastic pathways. Leahy et al [18] showed that *Methanobrevibacter* could convert H_2_ and/or formate to CH_4_ via the hydrogenotrophic methanogen pathway. A similar study reported that *Methanobrevibacter ruminantium* is a rod-shaped bacterium with variable motility that is able to utilize hydrogen (H_2_), carbon dioxide, and formate as substrates for methane production [19]. Furthermore, *Methanobrevibacter* was reported as the major protozoa-associated methanogen [20]. Ciliate protozoa play an essential role in the interspecies hydrogen transfer and methane formation in rumen, which produce large mounts of H_2_ by hydrogenases. M*ethanomassiliicoccus* can use methylamine substrates for methanogenesis by H_2_-dependent methylotrophic pathway [21]. It has been reported that *Methanomassiliicoccus* was 1.5-fold more abundant in low CH_4_ emitters than in high CH_4_ emitters among dairy cows [22]. Wang et al [9] found that replacement of forage fiber with non-forage fiber sources would reduce methanogenesis in rumen by decreasing the relative abundance of *Methanobrevibacter* and increasing that of *Methanomassiliicoccus*. Therefore, the results indicated that grape seed procyanidin has significant effects on the populations of major methanogen communities that are closely associated with methane production.

### Effects of grape seed procyanidin on volatile fatty acid production and related changes in the rumen microbial composition

The changes in VFA production in response to grape seed procyanidin treatment are possibly associated with changes in the microbial population. It is well documented that acetate and butyrate accompany H_2_ production, which can be used for the CH_4_ formation by the methanogenic archaea, and that CH_4_ is positively associated with the acetate to propionate ratio [23]. Janssen [24] reported that increasing propionate formation was strongly associated with a decrease in CH_4_ production. In the present study, GSPE caused a significantly increased proportion of propionate, while the proportion of acetate tended to decrease. The RT-PCR results showed that the relative populations of methanogens and *Fibrobacter succinogenes* were significantly decreased by grape seed procyanidin treatment. Methanogens were the sole producers of methane in rumen, and the number of them was considered as the major driver of methane emission [25]. *Fibrobacter succinogenes* was the dominant fibrolytic bacteria in rumen, and the decreased relative abundance indicated that the addition of GSPE presented somewhat detrimental effects on the ability of degrading substrate. Protozoa are associated with the abundance of H_2_-producing microorganisms, with an important role in methane production [26]. The RT-PCR results also showed a significant decrease in the protozoal population upon the addition of GSPE. Jolazadeh et al [27] found that supplementation with GSPE decreased total protozoal population and improved the growth performance of Holstein bulls, which was in accordance with our results. Protozoa, as the major butyrate producers in the rumen, are positively associated with butyrate production and are also known to contribute to fiber degradation, which might also be responsible for the change in fermentation. Further analysis of the response of protozoa to grape seed procyanidin with regard to the inhibition of methanogens may aid in elucidating the role of this compound in rumen fermentation. Additionally, the pH is known to have a substantial effect on the microbial community structure and diversity in the rumen ecosystem [28]; specifically, the pH was significantly increased by the GSPE treatment in the present study. Moreover, previous studies reported that polymeric procyanidins play an important role in improving rumen metabolism and ruminant nutrition, including reducing protein degradation and inhibiting methanogenesis [29]. Hence, the decrease in methane production may also be explained by the fact that grape seed procyanidin affected the pattern of fermentation by shifts in the microbial populations. However, further research is needed to fully understand the *in vivo* activity of grape seed procyanidin in rumen microbial ecosystems.

## CONCLUSION

In conclusion, the data presented herein show that GSPE resulted in decreased methane emissions because the properties of GSPE had a profound effect on the archaeal community structure and shifted the fermentation pattern, which was explained by the differences in the abundances of *Methanobrevibacter* and *Methanomassiliicoccus*. Additionally, a decreased utilization of hydrogen for the methane producers and an increased consumption of hydrogen substrate from CH_4_ to propionate formation were observed. These findings suggest that grape seed procyanidin can be effective for modulating rumen fermentation and mitigating methane emissions. However, further *in vivo* research is needed to confirm the mechanism of low methane emission and the negative impact of grape seed procyanidin on rumen fermentation and ruminant metabolism.

## Figures and Tables

**Table 1:** Composition and nutrient levels of the substrate (% DM basis)

Items	Experimental substrate
Ingredient (% DM)	
Alfalfa hay	18.59
Corn silage	25.65
Steam-flaked corn	26.02
Soybean meal	7.43
Cottonseed meal	7.43
Beet pulp	5.58
Corn DDGS	7.43
Premix1)	1.86
Chemical composition (% DM)	
CP	16.4
RDP (% of CP)	56.3
NDF	34.2
ADF	23.0
NFC2)	39.3
Starch	24.9
NE_L_3) (Mcal/kg)	1.53

DM, dry matter; DDGS, distillers dried grains with solubles; CP, crude protein; RDP, ribosomal database project; NDF, neutral detergent fiber; ADF, acid detergent fiber; NFC, nonfiber carbohydrates; NE_L_, net energy for lactation.

1) Premix composition per kilogram: 1,230 mg of Cu (minimum [min]), 4,950 mg of Zn (min), 1,760 mg of Mn (min), 50 mg of I (min), 61 mg of Se (min), 37 mg of Co (min), 504,800 IU of vitamin A (min), 88,800 IU of vitamin D_3_ (min), and 2,100 IU of vitamin E (min), 700 mg of vitamin B_3_ (min).

2) Calculated as 100–(% NDF+% CP+% ether extract+% ash).

3) NE_L_ was estimated as described by the NRC [30].

**Table 2:** Effect of grape seed procyanidin on gas production and rumen fermentation parameters *in vitro* (n = 9)

Items	Treatment1)		SEM	p-value

	GSPE	CON		
Gas production (mL)	83.33**	112.44	3.74	0.01
DM digestibility (%)	55.4**	67.8	1.57	0.01
CH_4_ (mg/d)	7.27**	9.97	0.35	0.01
H_2_ (μg/d)	7.87	7.31	0.46	0.56
pH	6.72**	6.61	0.01	0.01
Total VFA (mM)	62.93*	67.88	1.13	0.02
Molar proportion				
Acetate	62.58	63.03	0.12	0.09
Propionate	22.89*	21.55	0.29	0.04
Butyrate	11.44	12.55	0.32	0.1
Iso-butyrate	0.74	0.69	0.02	0.15
Valerate	1.08	0.98	0.02	0.08
Iso-valerate	1.27	1.19	0.04	0.31
Acetate/propionate	2.74*	2.94	0.04	0.02

SEM, standard error of the mean; DM, dry matter; VFA, volatile fatty acid.

1) GSPE, treatment group, 1 mg/bottle grape seed procyanidin extract addition; CON, control group, no grape seed procyanidin extract addition.

* p<0.05;

** p<0.01: values followed by superscripted asterisks differed within different treatments.

**Table 3:** Effects of grape seed procyanidin on microorganism relative populations *in vitro*

Items	Treatment1)		SEM	p-value

	GSPE	CON		
Methanogens	1.04**	2.18	0.16	<0.01
Ciliate protozoa	2.17**	3.75	0.22	<0.01
Anaerobic fungi	3.62**	1.81	0.25	<0.01
*Fibrobacter succinogenes*	0.86**	1.75	0.12	<0.01
*Ruminococcus albus*	0.63	0.54	0.02	0.18
*Ruminococcus flavefaciens*	11.36	11.11	0.08	0.12

SEM, standard error of the mean.

1) GSPE, treatment group, 1 mg/bottle grape seed procyanidin extract addition; CON, control group, no grape seed procyanidin extract addition.

* p<0.05;

** p<0.01: values followed by superscripted asterisks differed within different treatments.

**Table 4:** Effects of grape seed procyanidin on alpha diversity indices of archaea *in vitro* (n = 9)

Items	Treatment1)		SEM	p-value

	GSPE	CON		
Sobs	245.17	255.50	32.33	0.88
Shannon	2.16	1.91	0.13	0.34
Simpson	0.22	0.33	0.04	0.14
ACE	350.42	371.96	31.04	0.75
Chao	333.35	347.98	32.78	0.84
Coverage	0.998	0.998	0.000	0.73

SEM, standard error of the mean; ACE, abundance-based coverage estimator.

1) GSPE, treatment group, 1 mg/bottle grape seed procyanidin extract addition; CON, control group, no grape seed procyanidin extract addition.
